# Predictors of post traumatic growth in allogeneic hematopoietic stem cell transplantation survivors: a cross-sectional survey

**DOI:** 10.1186/s40359-023-01204-4

**Published:** 2023-08-16

**Authors:** Gemma McErlean, Caley Tapp, Lisa Brice, Nicole Gilroy, Masura Kabir, Matt Greenwood, Stephen R Larsen, John Moore, David Gottlieb, Mark Hertzberg, Louisa Brown, Megan Hogg, Gillian Huang, Christopher Ward, Ian Kerridge

**Affiliations:** 1 School of Nursing, Faculty of Science, Medicine and Health, University of Wollongong, Wollongong, NSW Australia; 2 Illawarra Health and Medical Research Institute, University of Wollongong, Wollongong, NSW Australia; 3 School of Public Health, University of Queensland, Herston, QLD Australia; 4 Queensland Centre for Mental Health Research, Wacol, QLD Australia; 5 Department of Haematology, Royal North Shore Hospital, Sydney, NSW Australia; 6 Centre for Infectious Diseases and Microbiology, Westmead Hospital, Sydney, NSW Australia; 7 Westmead Breast Cancer Institute, Sydney, NSW Australia; 8 Northern Blood Research Centre, Kolling Institute, University of Sydney, Sydney, NSW Australia; 9 Institute of Haematology, Royal Prince Alfred Hospital, Sydney, NSW Australia; 10 Department of Haematology, St Vincents Hospital, Sydney, NSW Australia; 11 Department of Haematology, Westmead Hospital, Sydney, NSW Australia; 12 Faculty of Medicine and Health Sciences, University of Sydney, Sydney, NSW Australia; 13 Department of Haematology, Prince of Wales Hospital, Sydney, NSW Australia; 14 Department of Haematology, Calvary Mater Hospital, Newcastle, NSW Australia

**Keywords:** Bone marrow transplant, Stem cell transplant, Hematopoietic stem cell transplantation, Allogeneic transplant, Post traumatic growth, Cancer, Survivors, Survivorship, Cancer care

## Abstract

**Aims:**

Given the increasing number of Hematopoietic Stem Cell Transplantations (HSCT) performed world-wide, the increasing likelihood of survival following HSCT, and the profound physical, psychosocial, and emotional impact of HSCT on survivors, their carers and families, it is important to identify factors that may contribute to or support post-traumatic growth (PTG) after transplant. In this study, we aimed to investigate the prevalence of PTG in an Australian cohort of long-term allogeneic HSCT survivors and describe associations between PTG and relevant clinical, sociodemographic and psychological variables.

**Methods:**

This was a large, multi-centre, cross sectional survey of Australian HSCT-survivors inviting all those transplanted in New South Wales between 2000 and 2012. Respondents completed the PTG Inventory (PTGI), the Sydney Post-BMT Survey, FACT-BMT, DASS 21, The Chronic Graft versus Host Disease (GVHD) Activity Assessment–Patient Self-Report (Form B), the Lee Chronic GVHD Symptom Scale, and the Fear of Cancer Recurrence Scale. Data was analysed using independent t-tests, one-way analysis of variance, and pearson’s correlations, and hierarchical multiple regression adjusted for potential confounders and to ascertain independent associations of explanatory variables with PTG.

**Results:**

Of 441 respondents, 99% reported some level of PTG with 67% reporting moderate to high levels of PTG. Female gender, younger age, complementary therapy use, anxiety, psychological distress and psychosocial care, and higher quality of life were associated with higher levels of PTG. Importantly, we also found that PTG was not associated with either chronic GVHD or post-HSCT morbidity.

**Conclusions:**

In this study – the largest study of PTG in long-term allogeneic HSCT survivors - we found that growth appears ubiquitous, with 99% of survivors reporting some degree of PTG and 67% reporting moderate-high levels of PTG. Importantly, we found no association with GVHD or chronic physical post-HSCT morbidity, or adverse financial, occupational or sexual impacts. This suggests that it is the necessity for and experience of, HSCT itself that foments personal growth. Accordingly, healthcare professionals should be alert to the profound and wide-ranging impact of HSCT - and the degree to which survivor’s may experience PTG. Identifying interventions that may assist HSCT survivors cope and building their resilience is of utmost importance.

## Background

Allogeneic Hematopoietic Stem Cell Transplantation (HSCT), also known as Blood and marrow transplant or bone marrow transplant (BMT), is a high-risk but potentially life-saving medical procedure that has a profound and pervasive impact on transplant recipients. Performed for more than 30,000 malignancies, haematological and autoimmune diseases worldwide per year (in 2018) [1, 2], it impacts on survivors physically, socially, economically, spiritually and psychologically and requires multifaceted life-long follow-up and support for recipients and their care givers [3]. In Australia the Australasian Bone Marrow Transplant Recipient Registry (ABMTRR) report over 80% one-year survival rates (for all allogeneic BMTs) and up to 69% for ten-year survival rates (dependent on primary disease and donor type) [4]. While it has been described as a ‘traumatic event’ with high rates of anxiety, depression, post-traumatic stress and decreased quality of life (QoL) reported by survivors and their families [5–9], personal growth and psychological recovery may be possible [10, 11].

Post traumatic growth (PTG) has been defined as, “the experience of positive psychological change, reported by an individual as a result of the struggle with trauma or any extremely stressful event” [12]. According to Tedeschi, Park, & Calhoun [13] a positive reappraisal of trauma can lead to changes including an increased appreciation for life, enhanced relationships and connectedness to others, increased personal strength, and deepened spirituality. It has also been found to result in less emotional distress, enhanced QoL and better physical wellbeing [14, 15]. Growth is thought to be the consequence of the challenge to previously held beliefs through a process of reflective functioning which results in a cognitive restructuring of beliefs and assumptions about the world.

For more than twenty years PTG has been studied in survivors of solid organ transplantation and patients who have been treated for serious chronic illness including heart disease, HIV and cancer [15–17]. PTG in these studies were associated with female gender, more perceived social support (including household management, transport and financial support) [18–22], access to and use of nurse counselling, greater time lapse since diagnosis/transplant, perceived stress and intensity of treatment received, advanced stage of cancer, better problem solving abilities, religious faith, and a strong sense of group belonging [18–22]. Those with lower self-reported general health status have been found to have significantly lower PTG [19]. Studies in patients with cancer have found no relationship between PTG and cancer site, cancer surgery, cancer recurrence, psychological distress or well-being, or having opportunity to discuss ones cancer diagnosis or treatment [22]. Associations between post PTG, education and age are inconsistent [22, 23].

In studies of allogeneic (donor) and autologous (self) HSCT survivors, PTG has been associated with female gender, younger age, higher educational levels, support from healthcare professionals, utilisation of nurse counselling, intrusive thinking, frequency of religious activities, optimism, perceived social support, problem solving abilities, using rewards as a coping strategy prior to transplantation and high pre and acute transplant distress levels [11, 22, 24]. However, these studies are small, include heterogeneous groups, and none have been conducted in an Australian cohort.

The increasing number of HSCTs performed world-wide, coupled with the increasing likelihood of survival following HSCT, has heightened recognition of the profound physical, psychosocial, and emotional impact of HSCT on survivors, their carers and families. This impact is principally a consequence of the deleterious effects of graft versus host disease (GVHD) and the treatment needed for GVHD; a condition which occurs in up to 70% of long-term survivors whereby the t-cells in the donor haematopoietic stem cell graft recognise the host (the patient’s) cells as foreign and initiate an immunological response which can cause significant and, in some cases, irreparable damage to every body system [25, 26]. Therefore, it is important to identify factors that may contribute to, or support PTG in this population. In this study we report the prevalence of PTG in an Australian cohort of long-term allo-HSCT survivors and describe associations between PTG and relevant clinical, sociodemographic and psychological variables.

## Methods

### Participants and procedures

This study reports results from a larger cross-sectional survey project which assessed the health, financial, cognitive, sexual and psychological experience of life post-transplant of 441 allo-HSCT survivors [26–30]. The study sample was selected from allogeneic transplant databases of four major metropolitan hospitals in New South Wales (NSW), Australia (at the time of study conception there were four transplant centres in NSW so this study included 100% of transplant sites; there are now five). Participants were eligible if they were > 18 years of age and had undergone an allogeneic BMT between 1st January 2000 and 31st December 2012, could read and write English and provide written consent. Those who had relapsed at the time of survey distribution were excluded. Survivor’s names and phone numbers were provided by the BMT Departments (BMT Co-ordinators and Data Managers) to the research team and eligible participants were phoned or approached when attending their HSCT clinic. Consenting participants were given the option to self-complete the survey or to complete with one of the researchers via a phone interview. A reminder telephone call was made to consenting participants who had not returned the survey within a month. All authors had access to primary clinical trial data.This study was approved by the Northern Sydney Local Health District Research Ethics Committee (NSLHD Reference: 1207–217 M.

### Instruments

Participants completed a 20-page questionnaire titled The Sydney Post BMT Study survey (SPBS) which included a questionnaire uniquely developed by the research team and 6 other instruments previously validated in allo-HSCT and other cancer populations. These included the Post Traumatic Growth Inventory (PTGI) [12], the Depression Anxiety and Stress Scale (DASS21) [31], the Fear of Recurrence Scale [32], the Functional Assessment of Cancer Therapy – Bone Marrow Transplant (FACT-BMT Version 4) [33], the Chronic GVHD Activity Assessment – Patient Self Report (Form B) [34], and the Lee Chronic GVHD Symptom Scale [35]. In total this took approximately one hour to complete.

### The post traumatic growth inventory (PTGI)

The PTGI is a 21 item questionnaire which measures post traumatic growth experiences in trauma survivors’ lives [12]. Tedeschi and Calhoun [12] identified five major domains of growth which include (1) greater appreciation of life and changed sense of priorities; (2) warmer, more intimate relationships with others; (3) a greater sense of personal strength; (4) recognition of new possibilities or paths for one’s life; and (5) spiritual development. It is widely used to assess positive life changes following traumatic events such as cancer, HIV, rape and disasters and other crises. Statements including ‘I developed new interests’, ‘I know that I can handle difficult situations’ and ‘I learned a great deal about how wonderful people are’ expressed and the reader is asked to respond using a six-point Likert scale with responses ranging from, ‘I did not experience this change’ to ‘I experienced this change to a very great degree as a result of my crisis’. The total of all 21 items yields a growth score which ranges from 0–105. Higher scores are indicative of greater growth. For the purposes of analysis survivors with a score of 0 experienced no PTG, 1–42 very small or small degrees of PTG, 43–63 moderate degrees of PTG and scores > 64 great or very great degrees of PTG.

### The depression anxiety and stress scale (DASS21)

The DASS 21 is a 21 item self-report questionnaire designed to measure the severity of a range of symptoms common to both depression and anxiety [31]. It is widely used and has been shown to have good inter-rater reliability, test-retest reliability and validity in both non-clinical and clinical cohorts [36–39]. In the current sample, internal consistency was excellent (Cronbach α = 0.93). Patients are asked to indicate how much a particular statement has applied to them over the past week using a four-point Likert scale ranging from ‘did not apply to me’ to ‘applied to me very much, or most of the time’. The total of all 21 items provides a score ranging from 0 to 63. Higher scores are indicative of greater symptoms of psychological distress.

### The functional assessment of cancer therapy – bone marrow transplant (FACT-BMT version 4)

The FACT-BMT is a validated questionnaire for measuring Quality of Life (QoL) in BMT recipients [40]. It takes three to five minutes to complete and combines two instruments, the FACT-G (FACT- General) and a BMT subscale. The FACT-G is a 28-item self-report instrument that measures QoL in cancer patients [33]. It consists of five subscales measuring physical, functional, social and emotional well-being and satisfaction with the doctor/patient relationship. The BMT subscale includes twelve items specifically designed for BMT patients. The FACT-BMT plus the BMT subscale provides an overall QoL score. Patients rate themselves over the past seven days using five-step Likert scales with responses used to calculate overall QoL and subscale wellbeing scores. The FACT-BMT is a reliable and valid measure, that has demonstrated sensitivity to clinical significant change [40]. In the current sample, internal consistency was excellent (Cronbach α = 0.94). The scores for FACT-G and FACT-BMT range from 0 to 148. Higher scores indicate higher QoL.

### The chronic GVHD activity assessment – patient self report (form B)

The Chronic GVHD Activity Assessment – Patient Self Report Form B was developed by the National Institute of Health (NIH) Consensus Development Project [34]. It is a ten-item questionnaire which asks patients to report on the severity and intensity (out of 10) of skin, oral, ocular and vulvovaginal symptoms as well as perceived global ratings of GVHD. It takes about one minute to complete. For the purposes of the analysis PTG was correlated with (a) survivors reported global rating of GVHD severity (none-mild-moderate-severe) and (b) survivors reported severity score (0–10).

The remaining instruments included questions about demographics, medical complications, referrals/investigations, pharma and non-pharmacotherapy, oral/dental health, infections, vaccinations, complementary therapy use, cancer screening, travel history, close personal contacts, lifestyle, nutrition, infection risk, work status, fertility and sexual function, relationships, long-term follow-up care, psychosocial concerns and a qualitative question ‘What are the three things that have impacted you most?’. The questionnaire used tick box response, short answer questions and 5-step Likert Scale measuring attitudes and other factors. The questionnaire was piloted in clinic and phone interviews to assess face and content validity and to check for comprehension of the survey questions.

Data was also collated from transplant databases on diagnosis, disease status and date of transplantation, conditioning regimen, GVHD prophylaxis, stem cell source and donor type for each consenting participant.

### Statistical analysis

Categorical responses were summarised using frequencies and percentages. Continuous variables were summarised using means and standard deviations. Two sample comparisons of parametric data were determined using independent t-test. Comparisons of greater than two samples were determined using one-way analysis of variance. Pearson’s correlations were utilised to examine associations between predictors of interest. Hierarchical multiple regression analyses were used to adjust for potential confounders and to ascertain independent associations of explanatory variables with outcomes of interest.

A two-tailed p value < 0.05 was used as the level of statistical significance. Statistical analysis was performed using Stata software (Version 16.1).

## Results

**Fig. 1:**
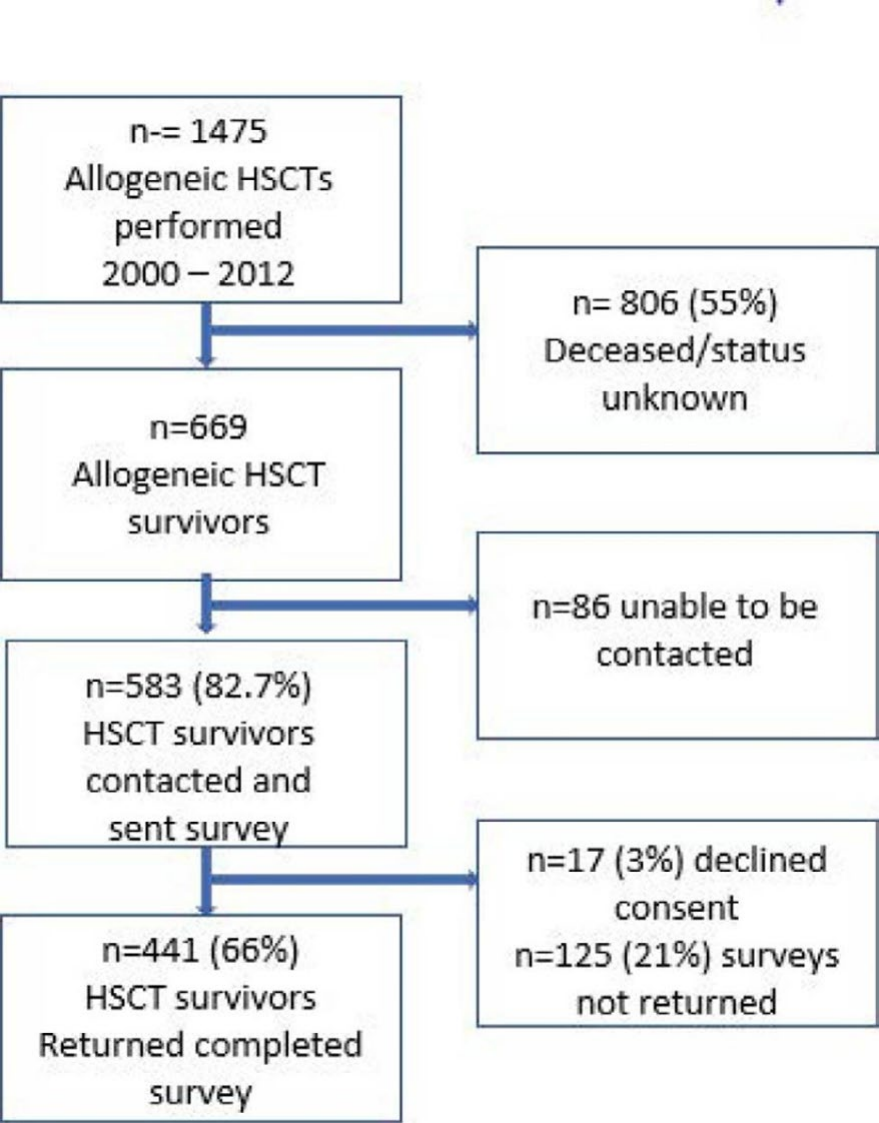
Study flowchart

A total of 1,475 Allogeneic HSCT were performed in the study period across all major transplant centres in NSW, Australia (all centres at the time of survey distribution). Four hundred and forty-one HSCT survivors (66% of total eligible, 76% of those contacted) returned the completed survey, 3% explicitly declined (Fig. 1). Of those completing the survey, 250 (57%) were male and 191 (43%) were female, and median age was 54 years (Range: 19–79). The median time since transplant was 5 years (Range: 1–14) and chronic multi-morbidity was common; iron overload (32.5%), osteoporosis/osteopenia (29.1%), hypertension (28.9%), cataracts (28.9%), depression (23.3%) and anxiety (20.6%), and secondary cancers were diagnosed in 24%. Chronic GVHD was prevalent and was reported by 69.3% of respondents. Almost half were university educated, and low, middle and high household income was approximately evenly distributed across the cohort. Most (over 70%) lived in metropolitan areas and almost 80% were in a relationship. Approximately half of our respondents received high-dose, myeloablative conditioning (48.7%) and had a sibling donor (56.95%) (Table 1).

**Table 1:** Characteristics of survey participants (n = 441)

Participant Characteristic	n (%)
Gender	
Male	250 (56.69%)
Female	191 (43.31%)
Time since transplant (years), M (SD)	5.83 (3.45)
Age	
Age in years at time of survey, M (SD)	52.17 (12.64)
Age in years at date of transplantation, M (SD)	46.27 (12.98)
Education	
University (some/completed) Other	154 (46.25%) 179 (53.75%)
Occupational status	
Employed Other^a^	209 (47.39%) 203(46.03%)
Household income, post-transplant	
Low income $20,000-$39,999 Middle income $40,000-$79,999 High income >=$80,000	155 (36.64%) 123 (29.08%) 145 (34.28%)
Residential location	
Major city Other (regional, remote)	311 (72.16%) 120 (27.84%)
Relationship status	
Married/De facto Single/Divorced or separated	344 (79.26%) 90 (20.74%)
Donor type	
Sibling related Matched Unrelated Haploidentical/Mismatched	250 (56.95%) 158 (35.99%) 31 (7.06%)
Conditioning regimen	
Myeloablative Reduced Intensity	214 (48.75%) 225 (51.25%)
Post Traumatic Growth Score, M (SD)	49.48 (21.41)
DASS, M (SD)	26.27 (23.61)
FACT-BMT, M (SD)	106.06 (21.47)
Chronic GVHD self-reported severity, M (SD)	3.51 (2.91)
Number parts of body affected by GVHD, M (SD)	2.15 (2.12)
Number of comorbidities	2.45 (1.98)

^a^ Casual, homemaker, unemployed, unable to work, retired

### Post traumatic growth

Almost all survivors of HSCT experienced some degree of PTG, with only 1% reporting no PTG. Thirty two percent of survivors reported small degrees of PTG, 40% reported moderate PTG and 27% reported great to very great degrees of PTG. PTG occurred in all five domains described in the PTGI with mean scores as follows: relating to others (*M* = 20.52, *SD* = 8.83), new possibilities (*M* = 9.38, *SD* = 5.64), personal strength (*M* = 10.86, *SD* = 5.53), spiritual change (*M* = 3.19, *SD* = 3.34), appreciation of life (*M* = 5.52, *SD* = 2.75).

### Demographic and transplant associations with Post traumatic growth

Mean PTGI scores differed between men and women, and according to age. PTG scores were significantly higher for women (*M* = 54.40, *SD* = 20.85) compared to that of men (*p* < .001). Participants who were aged under 54 years had significantly greater PTG (*M* = 53.24, *SD* = 19.15), compared to those who were older than 54 years (*M* = 45.88, *SD* = 22.83) (*p* < .001). People who had used any complementary therapy had higher PTG scores (*M* = 52.48, *SD* = 20.20) compared to people who had used none (*M* = 46.05, *SD* = 22.26) (*p* = .002). PTGI scores were not found to differ across socioeconomic variables, transplant or clinical factors or lifestyle factors such as BMI, travel or resumption of sex post-HSCT (Table 2). Zero-order correlations showed a similar pattern, with both being male and age having statistically significant negative correlations with PTGI scores. In addition, seeing a psychologist was found to be significantly positively correlated with PTGI scores, as were DASS scores. Gender (male), age, depression, anxiety and stress, and QoL were significantly negatively correlated with one another (see Table 3). PTG did not differ according to number of comorbidities (either as a continuous score representing number of comorbidities or when categorised as at least one comorbidity vs. no comorbidities).

**Table 2:** Demographic, social, transplant variables and their associations with post traumatic growth

Variables	Mean PTGI (SD)	P value
Demographic variables		
Gender		
Male (n = 246) Female (n = 188)	45.71 (21.10) 54.40 (20.85)	***t*(432) = -4.27**, ***p*** **< .001**
Age (years)		
< 54 (n = 212) ≥ 54 (n = 222)	53.24 (19.15) 45.88 (22.83)	***t*(432) = 3.63**, ***p*** **< .001**
Postcode		
City-Metro (n = 305) Regional or remote (n = 119)	49.71 (21.08) 48.59 (22.43)	*t*(422) = -0.48, *p* = .69
Marital status		
Married or de facto (n = 339) Other ^a^ (n = 89)	50.06 (22.48) 48.86 (21.02)	*t*(426) = 0.47, *p* = .64
**Socioeconomic variables**		
Education		
University Education (n = 129) No University Education (n = 199)	49.79 (22.19) 48.90 (21.20)	*t*(326) = 0.36, *p* = .72
Post-transplant Income ^b^		
Low income (n = 153) Middle income (n = 120) High income (n = 144)	49.96 (22.48) 50.32 (21.58) 49.33 (19.56)	*F*(2, 414) = 0.07, *p* = .93
Occupational status		
Employed (n = 209) Other ^c^(n = 203)	49.76 (22.66) 49.42 (20.39)	*t*(403) = 0.16, *p* = .87
**Transplant factors**		
Years since transplant		
< 6 years (n = 226) > 6 years (n = 180)	50.57 (21.07) 48.74 (21.52)	*t*(404) = 0.86, *p* = .39
Conditioning		
Myeloablative (n = 209) Reduced Intensity (n = 223)	51.19 (20.36) 47.87 (22.34)	*t*(430) = 1.61, *p* = .11
**Clinical factors**		
Pretransplant cancer diagnosis		
Yes (n = 358) No (n = 59)	48.92 (21.13) 53.88 (21.07)	*t*(415) = 1.67, *p* = .10
Comorbidities		
Yes (n = 360) No (n = 74)	45.62 (23.81) 50.27 (20.83)	*t*(432) = -1.70, p = .09
GVHD		
Yes (n = 297) No (n = 130)	49.84 (21.98) 48.18 (20.18)	*t*(425) = 0.74, *p* = .46
Severity of GVHD^d^		
None (n = 41) Mild (n = 128) Moderate (n = 69) Severe (n = 27)	50.39 (23.34) 50.20 (21.38) 51.59 (18.95) 46.15 (19.81)	*F*(3, 261) = 0.44, *p* = .72
**Medical care**		
Time spent attending medical care		
≤ monthly (n = 311) ≥ monthly (n = 115)	50.11 (20.69) 48.28 (23.17)	*t*(424) = 0.78, *p* = .43
Complementary therapy use		
Yes (n = 231) No (n = 203)	52.48 (20.20) 46.05 (22.26)	***t*(432) = -3.15**, ***p*** **= .002**
**Lifestyle factors**		
BMI		
Healthy weight (n = 195) Not healthy weight (n = 204)	50.47 (21.24) 49.25 (20.92)	*t*(397)=-0.57, *p* = .57
Travel post-BMT		
Yes (n = 229) No (n = 197)	50.47 (20.56) 48.17 (22.25)	*t*(424) = 1.01, *p* = .27
Sex post-BMT		
Yes (n = 283) No (n = 130)	50.62 (21.00) 47.75 (21.15)	*t*(411) = -1.29, *p* = .20

*Note.*^a^ Single, divorced, separated. ^b^ Low income=$20,000-$39,999; Middle Income=$40,000-$79,999; High Income=$80,000+. ^c^ Casual, homemaker, unemployed, unable to work, retired. ^d^ Question asked only of those who indicated they had experienced GVHD.

**Table 3:** Intercorrelations between study variables and post traumatic growth

	1	2	3	4	5	6	7	8	9	10	11	12	13	14	15	16	17	18
1. PTGI total	-																	
2. Male	**-0.20**	-																
3. Age	**-0.17**	**0.13**	-															
4. Employed post-transplant	-0.01	0.04	-0.22	-														
5. University educated	-0.04	0.11	-0.09	**0.23**	-													
6. Years since transplant	-0.05	-0.04	0.02	**0.17**	-0.01	-												
7. Cancer	-0.08	0.02	-0.02	0.05	0.05	0.06	-											
8. Conditioning	-0.08	**0.11**	**0.45**	**-0.12**	0.01	**-0.23**	-0.05	-										
9. Chronic GVHD Activity Assessment	-0.03	**0.14**	0.06	**-0.13**	-0.05	-0.07	0.04	0.09	-									
10. Count GVHD	0.03	0.05	0.04	-0.07	**0.15**	0.06	0.07	-0.04	**0.48**	-								
11. GVHD Y/N	-0.04	-0.06	-0.07	0.00	-0.09	-0.05	-0.07	0.02	**-0.17**	**-0.68**	-							
12. Comorbidities Y/N	0.05	0.06	**0.22**	**-0.16**	-0.02	**0.31**	0.04	-0.01	**0.14**	**0.29**	**-0.22**	-						
13. Psychologist	**0.11**	-0.03	**-0.15**	**-0.17**	0.10	-0.04	-0.02	-0.05	0.02	0.08	0.00	**0.24**	-					
14. Psychiatrist	0.01	0.10	-0.07	**-0.11**	**0.13**	0.07	-0.04	0.00	0.12	**0.10**	**-0.10**	**0.23**	**0.33**	-				
15. Antidepressants	0.04	-0.01	-0.09	**-0.13**	0.01	0.01	0.02	-0.05	0.02	0.07	-0.05	**0.20**	**0.37**	**0.20**	-			
16. Antianxiety	0.06	-0.04	-0.01	-0.04	-0.04	-0.01	0.02	-0.03	0.12	0.08	-0.05	**0.19**	**0.21**	**0.10**	**0.48**	-		
17. Complementary therapy	**0.15**	-0.12	0.01	0.06	**0.17**	**0.14**	-0.01	-0.04	-0.06	0.04	-0.04	0.04	0.08	-0.01	0.02	-0.04	-	
18. DASS Total	**0.17**	**0.11**	-0.04	**-0.14**	-0.09	0.03	0.03	**-0.10**	**0.33**	**0.25**	**-0.13**	**0.28**	**0.27**	**0.22**	**0.25**	**0.27**	0.00	-
19. Total FACT	**0.04**	**-0.10**	0.02	**0.27**	-0.02	0.05	-0.08	0.08	**-0.45**	**-0.28**	0.09	**-0.28**	**-0.28**	**-0.26**	**-0.27**	**-0.20**	0.01	**-0.66**

*Note.* Bolded values *p < .05*

### Regression analysis

Hierarchical regression was used to examine demographic, transplant, and mental health treatment-related variables as well as QoL, and levels of psychological distress as predictors of PTG (Table 4). The order of predictor inclusion in the model was guided by our intention to examine the relationship between QoL and psychological distress after demographic, transplant and mental health treatment variables are controlled for. We used hierarchical regression to allow us to examine changes in prediction after each group of predictors was added to the model.

**Table 4:** Hierarchical multiple regression predicting post traumatic growth after bone marrow transplant

	Model 1				Model 2				Model 3				Model 4			
Variable	*b*	*SE*	*95% CI*		*b*	*SE*	*95% CI*		*b*	*SE*	*95% CI*		*b*	*SE*	*95% CI*	
Age	-0.31**	0.11	-0.52	-0.10	-0.32**	0.12	-0.55	-0.08	-0.30*	0.12	-0.54	-0.06	-0.28*	0.12	-0.52	-0.05
Male	-10.53***	2.53	-15.52	-5.54	-10.60***	2.56	-15.64	-5.56	-9.48***	2.62	-14.64	-4.31	-10.42***	2.56	-15.48	-5.37
Employed post-transplant	-1.76	2.66	-6.99	3.47	-1.63	2.71	-6.97	3.71	-0.52	2.83	-6.10	5.07	-2.05	2.81	-7.59	3.48
University educated	-2.38	2.60	-7.50	2.74	-2.33	2.62	-7.49	2.83	-4.07	2.67	-9.33	1.19	-1.68	2.67	-6.93	3.57
Years since transplant					-0.04	0.38	-0.79	0.71	-0.29	0.39	-1.06	0.48	-0.43	0.38	-1.18	0.32
Cancer					-1.78	3.71	-9.09	5.53	-1.92	3.68	-9.18	5.33	-1.57	3.59	-8.64	5.50
Conditioning					0.19	2.85	-5.42	5.80	-0.11	2.84	-5.71	5.49	-0.18	2.78	-5.66	5.30
Psychologist									4.19	3.45	-2.61	10.98	2.68	3.42	-4.05	9.41
Psychiatrist									5.00	5.07	-4.99	14.99	5.53	5.01	-4.35	15.40
Antidepressants									-3.38	5.04	-13.30	6.54	-3.91	4.93	-13.61	5.80
Antianxiety									4.46	5.81	-6.99	15.91	2.82	5.71	-8.42	14.06
Complementary therapy									6.42*	2.64	1.22	11.62	6.04*	2.57	0.97	11.10
DASS total score													0.29***	0.07	0.15	0.44
FACT BMT total score													0.23**	0.08	0.07	0.39
∆*R*^2^					0.001**				0.03				0.05***			
*R* ^2^	0.10				0.10				0.14				0.19			

*Note.* * *p < .05*, ** *p < .01*, *** *p* < .001

In the first step, age, sex (with female as the reference category), full- or part-time employment post-transplant, and university educated were entered and significantly predicted PTG, *F*(4, 251) = 7.26, *p* < .001. Treatment-related factors were entered in the second step, and *F* change indicated no significant improvement in prediction over the use of the socio-demographic variables alone, *F*(3, 248) = 0.08, *p* = .97. The addition of mental health treatment-related variables at step 3 made no significant additional contribution to the prediction of PTG, *F*(5, 243) = 1.96, *p* = .09. Across the first three models, age (model 3 *b*=-0.30, *p* < .05) and being male (model 3 *b* =-10.42, *p* < .001) individually made a significant contribution to the prediction of PTG. In terms of age, being older was associated with lower PTG and men had lower levels of PTG by comparison to women. Any use of complementary therapies was associated with higher levels of PTG when included in the third step(*b* = 6.42, *p* < .05). This pattern held for the final model. The addition of DASS and FACT-BMT measures in the final model significantly improved the prediction of PTG, *F*(2, 241) = 7.97, *p < .001*. After controlling for socio-demographic variables, treatment-related and mental health variables, a higher total DASS score, were significantly associated with higher levels of PTG (*b* = 0.29, *p* < .001). In addition, higher levels of QoL were also associated with higher levels of PTG (*b* = 0.23, *p* < .01). The number of years since transplant was not significantly associated with PTG in this analysis.

An additional regression analysis was conducted to examine the relationship between GVHD and PTG (Table 5). In an analysis of a sub sample of people who indicated they had experienced GVHD, the same regression model above was tested, with the exception that GVHD severity and count of GVHD symptoms were included in the second step alongside treatment-related factors. The pattern of results was almost identical to the main analysis reported above, with the exception that inclusion of GVHD-relevant variables resulted in age no longer being a significant predictor of PTG.

**Table 5:** Hierarchical multiple regression predicting post traumatic growth after bone marrow transplant in patients who had experienced GVHD

	Model 1				Model 2				Model 3				Model 4			
Variable	*b*	*SE*	*95% CI*		*b*	*SE*	*95% CI*		*b*	*SE*	*95% CI*		*b*	*SE*	*95% CI*	
Age	-0.26	0.15	-0.56	0.03	-0.19	0.17	-0.52	0.15	-0.04	0.18	-0.41	0.32	-0.08	0.18	-0.43	0.27
Male	-9.34**	3.48	-16.22	-2.47	-9.54*	3.69	-16.83	-2.25	-9.62*	3.73	-16.99	-2.24	-8.85*	3.68	-16.13	-1.57
Employed post-transplant	-5.01	3.66	-12.25	2.23	-4.16	3.82	-11.71	3.39	-0.92	4.05	-8.94	7.10	-2.85	3.98	-10.72	5.01
University educated	-2.49	3.49	-9.39	4.41	-2.29	3.66	-9.53	4.96	-3.77	3.74	-11.17	3.64	-1.83	3.68	-9.10	5.45
Years since transplant					-0.20	0.51	-1.20	0.80	-0.38	0.53	-1.44	0.67	-0.60	0.52	-1.63	0.44
Cancer					-0.78	5.69	-12.04	10.48	0.50	5.78	-10.93	11.94	1.47	5.61	-9.63	12.58
Conditioning					-4.14	3.88	-11.82	3.54	-5.50	3.90	-13.22	2.23	-6.00	3.80	-13.53	1.53
cGVH severity					0.45	0.83	-1.19	2.09	0.90	0.86	-0.81	2.61	0.89	0.89	-0.88	2.66
GVHD count					0.10	1.18	-2.22	2.43	-0.18	1.19	-2.54	2.17	0.46	1.18	-1.88	2.80
Psychologist									7.40	4.70	-1.89	16.68	6.19	4.71	-3.13	15.51
Psychiatrist									-0.52	6.40	-13.18	12.13	3.74	6.50	-9.12	16.60
Antidepressants									6.82	6.70	-6.44	20.08	5.11	6.64	-8.03	18.25
Antianxiety									-6.78	7.66	-21.94	8.38	-6.17	7.47	-20.95	8.61
Complementary therapy									5.23	3.62	-1.94	12.40	5.61	3.52	-1.35	12.57
DASS total score													0.30**	0.11	0.09	0.52
FACT BMT total score													0.36**	0.13	0.10	0.61
∆*R*^2^					0.01				0.05				0.06**			
*R* ^2^	0.09				0.11				0.15				0.22			

*Note.* * *p < .05*, ** *p < .01*, *** *p* < .00

## Discussion

This is the largest study of PTG in long-term allogeneic HSCT survivors. We found that PTG was almost universally experienced by our survivors; only 1% reported no PTG, and over two thirds reported moderate-high degrees of PTG post-HSCT, higher than that reported in the only meta-analysis of PTG (67% vs. 52.6% [41]). Importantly, we also found that PTG was not associated with either chronic GVHD or post-HSCT morbidity, suggesting that it is the necessity for and experience of, HSCT itself that foments personal growth. In other words, it is the experience of mortality salience (the experience one has of facing the possibility of their own death), that prompts reflection on one’s life and future and encourages PTG.

In our survivors, as with other smaller studies of PTG in HSCT survivors and patients treated for cancer, higher degrees of PTG were experienced by women, and those who were younger, reported higher anxiety and distress, used complimentary therapies, and had higher self-reported QoL [17, 22, 24, 42–44]. We also found that survivors who had received psychological care reported more PTG. The fact that women and younger people appear to experience greater degrees of PTG following HSCT is in many ways unsurprising. Women are disproportionately and more broadly impacted by many illnesses, including cancer, in every domain of life including, most recently, COVID-19 [45]. These impacts relate not simply to the illness itself or to a particular therapy, but to gendered economic, occupational and socio-political disadvantage, family and carer responsibilities and insufficient interpersonal and professional support. All of these factors would seem likely to lead female survivors of HSCT to reflect on their life, future and relational responsibilities.

Likewise, younger patients undergoing HSCT may be expected to have greater PTG as the experience of mortality salience (the risk of one’s own extinction) at a younger age is likely to be more confronting for a younger person than an older person who may have more health related and co-morbidity concerns [46]. For many young people undergoing HSCT, haematological malignancy is their only experience of serious illness, and the threat associated with HSCT is the first time that their future expectations and goals have been called into question. That this may prompt personal reflection and PTG seems self-evident.

The fact that those experiencing more anxiety and distress (as measured by DASS) also experienced greater degrees of PTG is consistent with other studies of PTG and is also consistent with the notion that PTG emerges from “within persons”, rather than from the event [47, 48].

Although the sample size and high response rate (76%) make it likely that these results represent an accurate account of PTG in allo-HSCT survivors in Australia, there are a number of limitations to our study that may limit the generalizability of these results to BMT survivors in other countries and other settings, and that may limit our understanding of PTG in HSCT. These limitations include participation, recall and misclassification bias, incomplete responses and restricted interference regarding casual or temporal relationships which are immutable in cross-sectional studies. Additionally, as this was self-report, we did not validate medical and other comorbidity or treatment data against medical records/hospital attendance/admissions. Importantly, our respondents were predominantly white/Caucasians (86.9%), which makes generalisability to other ethnic groups or cultural contexts difficult, not only because of the demographic differences, but also questions regarding the cross-cultural validity of the PTGI itself [49]. Finally, we did not ask about religious beliefs, spirituality and social support, nor did we ask carers about their experience with regard to PTG. This has been done elsewhere in cancer caregivers [46, 50] and is worthy of further study in this population given the arduous and life-altering nature of transplantation and long-term survivorship, which carers are intimately involved in and witness to.

The results of this and similar studies suggests that healthcare professionals should recognise the profound and potentially life-changing impact of HSCT for many patients, and acknowledge that HSCT may not only be physically challenging but transformative [51]. While it would be inappropriate for HSCT recipients to be told they “will grow” as a results of HSCT, or that it has positive psychological consequences, healthcare professionals should not minimise or normalise HSCT but communicate the ways in which HSCT may challenge every aspect of a patient’s life. Our results would also lend support to provision of expert psychosocial support pre and post-HSCT. This may include clinical psychologist referral, provision of informational resources and referral to support groups as desired. However, further research is required to identify the rates, extent and determinants of PTG in different cultures and ethnicities, to establish the temporal patterns of PTG post-HSCT and to identify what interventions may assist HSCT survivors and their partners and families cope with the complex disruptions posed by HSCT and become stronger and more resilient as a result.

## Conclusion and relevance for clinical practice

In this study – the largest study of PTG in long-term allogeneic HSCT survivors - we found that growth appears ubiquitous, and has no association with GVHD, chronic physical post-HSCT morbidity, or adverse financial, occupational or sexual impacts. This suggests that it is the necessity for and experience of, HSCT itself that foments personal growth. Accordingly, healthcare professionals should not minimise or normalise HSCT, but instead be alert to the profound and wide-ranging impact of HSCT - and the degree to which it may challenge every aspects of a survivor’s life and identity. HSCT is not only physically challenging, with wide-ranging and profound impacts, it is also, importantly, transformative. Identifying interventions that may assist HSCT survivors cope and building their resilience is of utmost importance.

This study adds important new information about the experience of survival following allogeneic-HSCT including exploration of sexual, financial, occupational and psychosocial impacts of transplant .The results of this research suggest that further work is necessary to develop and test interventions that may better prepare patients for transplant and improve their ability to cope with the significant impacts that transplant may have on their lives, and on the lives of those around them. Ultimately, this may lead to improvements in care provision and the quality of life of survivors.

## Data Availability

Datasets generated and/or analysed during the current study are not publicly available as the participants did not give consent for their raw data and transcriptions to be shared with other researchers outside of the research team, but may be available from the corresponding author upon reasonable request.

## List of Abbreviations

Allo-HSCT: Allogeneic haematopoietic stem cell transplantation
ABMTRR: Australia the Australasian Bone Marrow Transplant Recipient Registry
BMT: Blood and marrow transplant, bone marrow transplant
DASS: Depression Anxiety and Stress Scale
FACT-BMT: Functional Assessment of Cancer Therapy – Bone Marrow Transplant
FACT-G: Functional Assessment of Cancer Therapy - General
GVHD: Graft versus host disease (can be acute or chronic)
HSCT: Haematopoietic stem cell transplant
NIH: National Institute of Health
PTG: Post Traumatic Growth
PTGI: Post Traumatic Growth Inventory
SPBS: Sydney Post BMT Study Survey
QoL: Quality of Life
